# The Recruitment Niche Predicts Plant Community Assembly Across a Hydrological Gradient Along Plowed and Undisturbed Transects in a Former Agricultural Wetland

**DOI:** 10.3389/fpls.2019.00088

**Published:** 2019-02-06

**Authors:** Jose W. Valdez, Florian Hartig, Sabine Fennel, Peter Poschlod

**Affiliations:** ^1^Theoretical Ecology, Faculty of Biology and Preclinical Medicine, University of Regensburg, Regensburg, Germany; ^2^Ecology and Conservation Biology, Faculty of Biology and Preclinical Medicine, University of Regensburg, Regensburg, Germany

**Keywords:** agricultural land, community assembly, flooding, germination, land use, ontogenetic niche, regeneration niche, seed bank

## Abstract

Seedling emergence in plant communities depends on the composition in the soil seed bank, in combination with species-specific responses to the environment. It is generally assumed that this juvenile transition, known as the recruitment niche, is a crucial filter that determines species’ distributions and plant community assemblies. The relative importance of this filter, however, has been widely debated. Empirical descriptions of the recruitment niche are scarce, as most field studies focus on environmental effects at later life stages. In this study, we examine the importance of the recruitment niche for predicting plant communities across a hydrological gradient in a disturbed and undisturbed area in Lake Schmiechen, Baden-Württemberg, Germany. We combine a seed bank experiment, measuring germination in water basins under standardized conditions and different water levels, with field observations of plant communities along a hydrological gradient in plowed and undisturbed transects in a former agricultural wetland. We find that hydrology consistently predicted plant community composition in both the germination experiment and in the field. The hydrological recruitment niches measured in the seed bank experiment correlated with the hydrological niche in both the plowed and undisturbed area, with slightly stronger correlation in the plowed area. We explain the latter by the fact that the seed bank experiment most closely resembles the plowed area, whereas succession and competitive interactions become more important in the undisturbed area. Our results support the view that the recruitment niche is an important driver of species composition, in both the plowed and undisturbed area. Recognizing the recruitment niche and the response of seeds within a seed bank to environmental gradients and anthropogenic disturbance is necessary to understand and predict future plant community composition.

## Introduction

The niche, generally defined as the environmental conditions under which a species can maintain positive growth rates and persist (Hutchinson, 1957), is a central concept in ecology. It is widely assumed that species’ distributions and community composition are largely determined by the ecological niches of species and environmental gradients at the local and global scale (Wellborn et al., 1996; Pulliam, 2000; Pottier et al., 2013). However, the relative strength of niche effects compared to other factors, such as space or drift, are still debated (Vellend, 2010; Hubbell, 2011).

For any species, separate life stages may differ in their niche requirements, and environmental conditions may have different and complicated impacts on population viability (Grubb, 1977; Bond and Midgley, 2001; Bertrand et al., 2011). However, ontogenetic niches (the niche occupied by an organism during a specific life stage) are so far more commonly considered in animal ecology studies (Werner and Gilliam, 1984; Olson, 1996; Wellborn et al., 1996; Dopman et al., 2002; Subalusky et al., 2009), and research in plant ecology typically focuses on the adult life stages or assumes that life stages respond similarly to the environment (Clark et al., 1998; Sardinero, 2000; Collins and Carson, 2004; Crawley, 2009; Poschlod et al., 2013; Jiménez-Alfaro et al., 2016). However, it has been suggested that recruitment is a critical filter determining species distributions and communities since most plant mortality occurs during the transition from a seed to juvenile, and seed germination is strongly associated to environmental filters at local, regional, and global scales (Grubb, 1977; Pugnaire and Valladares, 1999; Young et al., 2005; Albrecht and McCarthy, 2009; Poschlod et al., 2013; Jiménez-Alfaro et al., 2016). The environmental conditions that allow a seed to germinate and establish has been called the recruitment niche (Young et al., 2005), and is one component of the broader regeneration niche, which includes successful reproduction and seed dispersal (Grubb, 1977; Reed and Clark, 1978; Young et al., 2005).

Successful recruitment requires the availability of seeds within the soil seed bank and environmental conditions which are suitable for the germination of viable seeds (Kelly, 2008; Poschlod et al., 2013; Jiménez-Alfaro et al., 2016). A basic filter for the presence of a species in a plant community is therefore the composition of the seed bank, which is dependent on seed dispersal and survival, and the species-specific environmental requirements for germination (Poschlod et al., 2013; Fraaije et al., 2015a,b). Although conditions such as temperature, light, and nutrients are critical for seeds to germinate and establish, water is often a major factor determining seed germination and the plant community which emerges from the seed bank (Poschlod et al., 2013; Fraaije et al., 2015b). Hydrological gradients have been shown to play a strong environmental filter for germination and seedling growth (Fraaije et al., 2015a,b), and are especially influential for determining community structure in aquatic habitats such as marshes (van der Valk and Davis, 1978; Baldwin et al., 2001) and (seasonal) wetlands (Keddy and Ellis, 1985; Casanova and Brock, 2000; Nicol et al., 2003; Peterson and Baldwin, 2004).

Besides germination of seeds within the seed bank, processes such as disturbance and succession may also influence community assembly. Plant communities in such areas are often found with a high proportion of long-term persistent seeds in the soil seed bank, allowing them to survive these regularly disturbed habitats (Poschlod et al., 2013). Disturbance from agricultural practices, such as drainage of aquatic habitats or plowing during dry periods can also have a significant effect on seed banks and plant communities (Bekker et al., 1997; Verhagen et al., 2001; Devictor et al., 2007; Bart and Davenport, 2015). Although plowing is generally thought to have a negative effect on community structure and species abundance (Bart and Davenport, 2015), opposite results have also been found. For example, in wetland communities, this disturbance does not necessarily kill the seeds, but can induce a spatial storage effect that favorably influences the species richness of seeds, seed abundance, and germination ability at the community level when compared to an undisturbed landscape (Devictor et al., 2007). Hydrological gradients also interact with interspecific interactions and competition to determine undisturbed community assemblages (Muñoz-Mas et al., 2017) and have been found to create differences in seed density and species diversity (Bekker et al., 2000). It is, therefore, necessary to consider the relationship between hydrological gradients and anthropogenic disturbance, such as plowing, to understand and predict the plant community that emerges and establish, which can be especially useful in the ecological restoration of previously disturbed sites.

The aim of this paper is to determine whether the recruitment niche predicts plant community composition in a former agricultural wetland controlling for the effects of hydrology in disturbed and undisturbed conditions. For this study, a seed bank and field experiment were undertaken to determine the hydrological recruitment niche for species within the soil seed bank and compared with the observed plant communities between a plowed and undisturbed area. We addressed two specific objectives: 1. Does water level influence germination for the species available within a seed bank? 2. Does a hydrological gradient in plowed and undisturbed areas correlate with the recruitment niche as determined in the seedbank experiment?

## Materials and Methods

### Study Area

**FIGURE 1:**
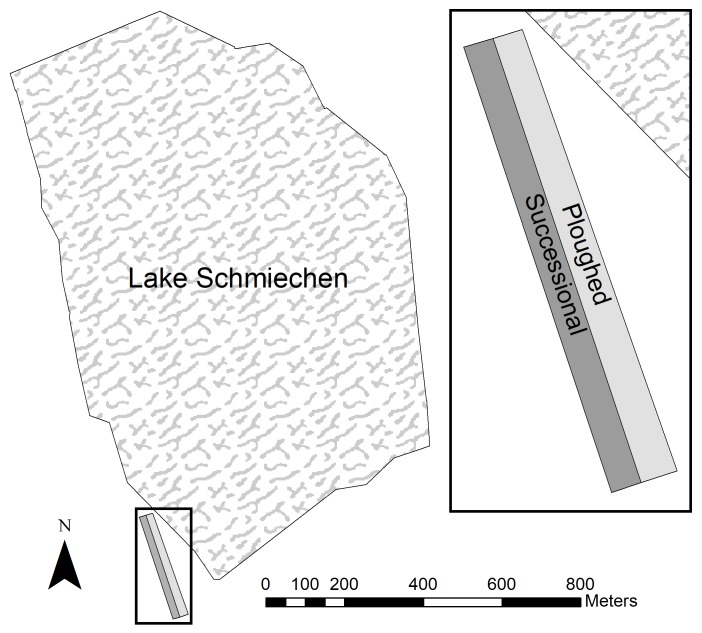
Former agricultural plot (plowed and undisturbed) in Lake Schmiechen, Baden-Württemberg, Germany.

The study site is located on former agricultural land at the south-western edge of Lake Schmiechen in Baden-Württemberg, Germany (Figure 1). This land was plowed since the Second World War and abandoned at the end of 1987. The area is situated on a layer of Jurassic strata clay and on the loop of the former “Ur-Danube.” During the spring and early summer, this shallow lake is characterized by seasonal flooding from rainfall and melting snow, with reduced water levels from late summer into winter. The field site (approximately 180 × 26 m), part of a former agricultural field, was situated parallel to the lake margins and risked with seasonal flooding. Due to increasing elevation from the northern to southern end, the field site exhibited a hydrological gradient as the flooding receded back into the lake.

The abandoned field site was covered by reeds with *Phalaris arundinacea* as the most dominant plant (cover between 40 and 80%), around 1.5 m high. Two other dominant wetland plants were *Agrostis stolonifera*, a creeping, flooding tolerant species (covering up to 50%) and *Eleocharis palustris*, also flooding tolerant with underground rhizomes (covering up to 25%). Two flood tolerant species indicating former arable field use were also very common – *Plantago major* (a rosette plant, cover up to 25%) and *Ranunculus repens* (creeping with aboveground stolons; cover also up to 25%).

### Seed Bank Experiment

Soil samples were taken before the beginning of the vegetation period in the first week of March 1997. The sampling took place in the middle of southern end of the field site in the middle of the transect. Soil samples were taken on a distance of 60 m. To collect a standardized seedbank for the experiment, 60 soil samples were taken at every meter in a transect approximately 15 cm × 15 cm and 10 cm deep (around 2.25 L). Soil samples were uniformly mixed and distributed in equal proportions in plastic pots (diameter 28 cm) to ensure the same initial floristic potential was present. The pots were first filled with 10 L of a sterile compost-sand mixture, over which 1.5 L of soil of the field site were applied in a thickness of about 2.5 cm. The experiment consisted of six hydrological treatments with ten replicates each, carried out as a common garden experiment in water basins of the Philipps-University of Marburg Botanical Garden under uniform light and temperature conditions similar like those at the Lake Schmiechen, while the water levels for each treatment remained constant. The water was similar as in Lake Schmiechen (slightly calcareous and mesotrophic), and treatment levels were controlled daily with an overflow drain. Hydrological treatments (10 replications per treatment) included permanently dry (water level approx. 20 cm below surface), permanently moist (water level near the surface), permanently flooded 10 cm above soil surface, and permanently flooded 40 cm above soil surface; as well as two temporal treatments, where pots were first flooded 40 cm above the soil surface, and then dried on either 15 June or on 15 August. The experiment lasted from mid of March until end of October. The number and species of germinating seeds from each sample were recorded and removed until no more seeds germinated.

### Field Experiment

The right half of the field site was plowed in spring 1991 and spring 1996 while the left area remained undisturbed to allow succession to occur (Figure 1). Each of the areas contained 47 permanent plots along transects bounded with metallic boundary marks (stones and nails) going from the southern to northern end. Surveying occurred yearly in summer from 1990 to 1999 (except 1993, 1997, and 1998). The plots had a size of 4 m^2^ and were situated along the transect every four meters. The elevation at each plot was recorded using a theodolite. Ground water table measurements were taken from a water gauge established by the water management office at the border of the lake. For each transect, we recorded species presence and percent cover using the Braun-Blanquet scale (Braun-Blanquet, 1964).

### Statistical Analysis

To determine the influence of the water level on species germination, we used non-metric multidimensional scaling (NMDS) ordination from the “vegan” R package (Dixon, 2003). NDMS is a nonparametric ordination method that is generally considered to be well-suited for ecological data due to its higher robustness compared to other ordination approaches (McCune and Mefford, 2005). Germination counts were log-transformed due to the large variability between species, and species with low total germination counts (three or less) were removed. In the NMDS, we analyzed the six treatments as unordered factors, and additionally numerically ordered according to their temporal hydrological level (1 = permanently dry, 2 = flooded above soil surface and dried in June, 3 = flooded above soil surface and dried in August, 4 = permanently moist (water level at the soil surface), 5 = permanently flooded 10 cm above soil surface, and 6 = permanently flooded 40 cm above soil surface). The ordered factors were then fitted as an environmental vector, with the direction of the vector arrow pointing to the direction of greatest change in the environmental variable and the arrow length representing the strength of the gradient (Oksanen, 2011). This allows a quick visual comparison if they cluster or align according to their water level.

As a second analysis, we fitted a hydrological recruitment niche optimum for each species, using generalized additive mixed models (GAMMs) from the “mgcv” R package (Wood and Wood, 2017). GAMMs are an extension of generalized linear models that allow fitting flexible functions for the relationship between predictor and response variables, which permits considering linear as well as more complex responses within the same model. Our base model contained log germination counts per sample as the dependent variable; with species and the interaction between species and the numerical treatment for the permanent water levels as the independent variables. We applied the smoothing function on the numerical treatment level per species and determined the optimum recruitment niche of each species where the curve had the highest predicted germination.

For the field experiment, we used NMDS to examine the relationship between community structure and environmental variables in the plowed and undisturbed halves of the field. The Braun-Blanquet cover abundance scale was converted to the midpoint of the cover range (Wikum and Shanholtzer, 1978). As predictors, we used elevation (meters above sea level) and annual groundwater levels as proxies for hydrology, and year as a proxy for undisturbed changes to the community structure.

**FIGURE 2:**
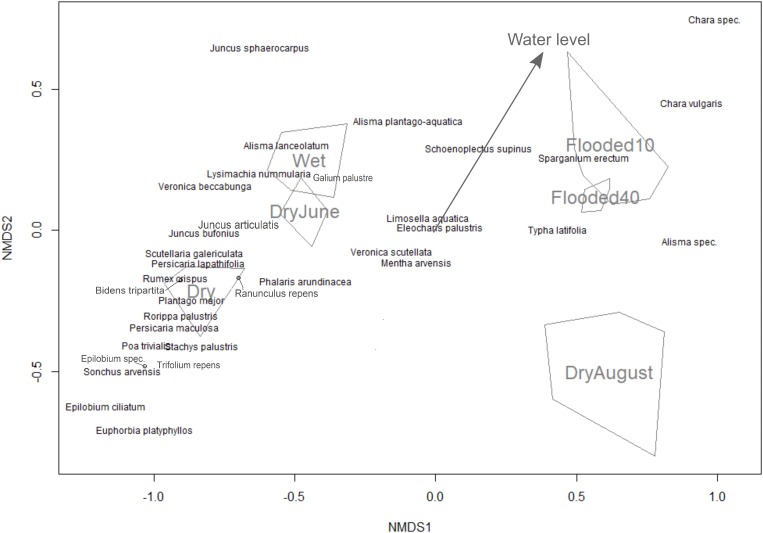
Non-metric multidimensional scaling ordination for the seed bank experiment with different water levels represented as a categorical variable (convex hulls) and fitted as an environmental vector (arrow).

To compare the seed bank and field experiment, as well as show consistency between NMDS and GAMM analysis, we calculated Spearman correlations between the niche optimum predicted for each species where the smoothing function was significant, and its value in direction of the hydrological axis of the NMDS [determined by rotating the NDMS ordination based on species positions projected on the water level vector (arrow in Figure 2)]. For the field transects, we simply used the first axis of the NMDS, because both hydrological variables (elevation and ground water level) were projected nearly exclusively on this axis. To compare significance between correlations, we used the Hittner et al. (2003) method using the “cocor” R package (Oberdorfer, 2001). All statistical analyses were conducted in program R (version 3.4.1).

## Results

### Seed Bank Experiment

Out of the 46 species that germinated in the seed bank experiment, 34 were observed more than three times. In the NMDS, the treatment of drying in June was clustered between permanently dry and permanently wet as expected, but the drying in August treatment was separate from the rest of the treatments (Figure 2). The drying in August treatment also had the lowest species diversity and germinating seeds compared to the other treatments (Table 1). The vector arrow from the metric treatment values indicated that the treatment as factors closely followed the assumed gradient, except for the flooding treatments which were clustered closely together, suggesting no differences between flooding at 10 and 40 cm (Figure 2). The best GAMM model predicting the number of germinated seeds was determined by the species and the treatment level. This model accounts for much of the variance in number of germinating seeds, with an adjusted R-squared of 82.6%. The smoothing terms from the model were significant for 27 out of the 34 species (Appendix S1).

**Table 1:** Comparison of species diversity and germinating seeds between hydrological treatments.

Treatment	Number of species	Average number of germinated seeds per sample (sd)	Total number of germinated seeds
Dry	25	4.41 (9.58)	1500
Dry June	25	6.91 (24.48)	2350
Dry August	8	1.17 (9.32)	583
Wet	25	6.92 (24.84)	2353
Flooded 10 cm	16	4.17 (14.11)	1419
Flooded 40 cm	12	2.17 (7.12)	2215

### Field Experiment

**FIGURE 3:**
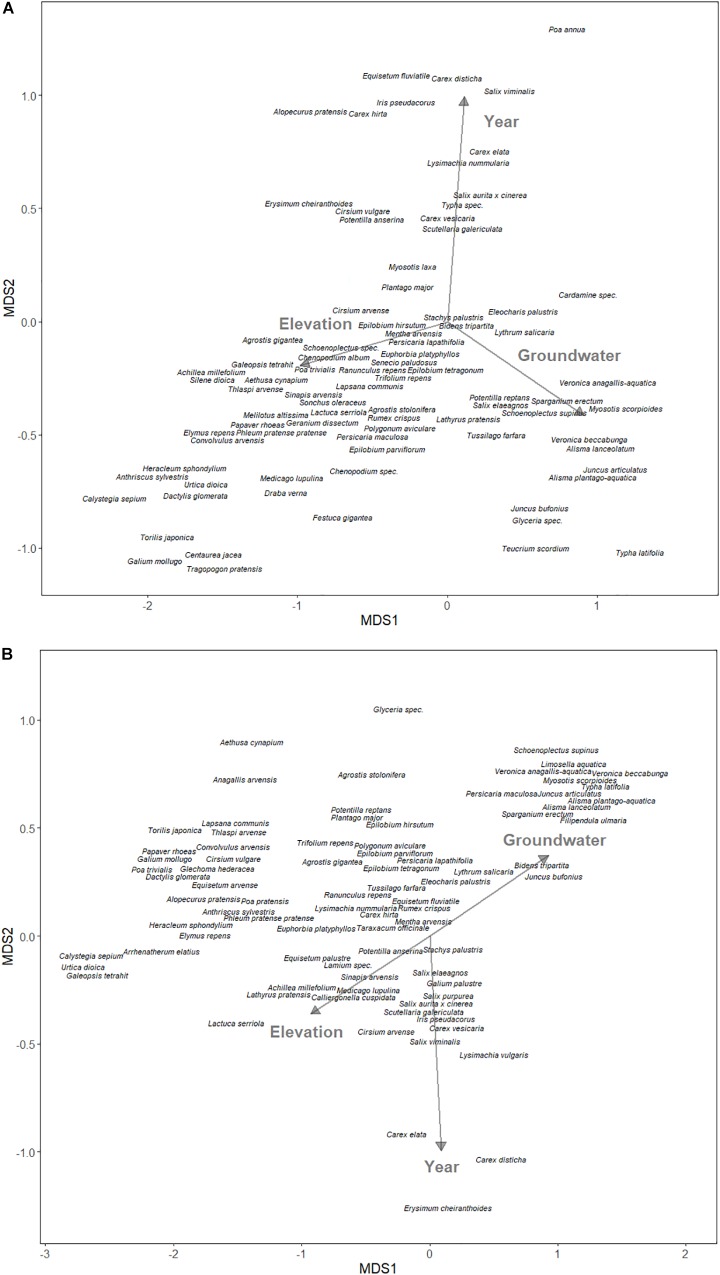
Non-metric multidimensional scaling ordination of species composition of **(A)** yearly plowed and **(B)** undisturbed site at the south-western edge of Lake Schmiechen in Baden-Württemberg, Germany. Environmental vectors are displayed with arrows.

One hundred and fourteen species were found in the aboveground vegetation of the field site, with 84 species in the plowed area, 82 species in the undisturbed area, and 68 species (80%) shared amongst the two areas. The NMDS projected the assumed water-related variables, elevation and groundwater level, mostly on the first NMDS axis, while the year was mostly on the second axis for both the plowed (Figure 3A) and undisturbed (Figure 3B) areas. The elevation was always opposite to groundwater level, which is expected when considering that greater elevation means reduced water levels.

### Seed Bank and Field Experiment Comparison

The niche optimum inferred from the GAMMs was strongly correlated with species positions along the hydrological axis of the NMDS using the seed bank data (Table 2), showing consistency between the two methods. The niche optimum was also significantly correlated with the species’ NDMS hydrological position in the field experiment for both the plowed and the undisturbed areas (Table 2), with the correlation coefficient higher in the plowed area compared to the undisturbed area (*z* = 1.8096, *p*-value = 0.0352). The NMDS from the seed bank experiment was significantly correlated with the hydrological position in both the plowed and undisturbed areas (Table 2). However, there was no difference between these correlations (*z* = 1.4448, *p*-value = 0.0743). The hydrological positions from the field NMDS were significantly correlated between the plowed and undisturbed areas (Table 2).

**Table 2:** Spearman’s rank correlation coefficient for species position based on the predicted niche from the GAMM, the hydrological axis of the NMDS in the seed bank experiment, and the hydrological axis of the NMDS in the plowed and undisturbed halves of the field experiment.

Variables		Correlation	*N*
GAMM niche optimum			
	Seed bank MDS1	0.88^∗∗^	27
	Plowed MDS1	0.76^∗∗^	18
	Undisturbed MDS1	0.59^∗^	20
Seed bank MDS1			
	Plowed MDS1	0.78^∗∗^	22
	Undisturbed MDS1	0.66^∗∗^	24
Plowed MDS1			
	Undisturbed MDS1	0.85^∗∗^	68

*^∗^p < 0.01, ^∗∗^p < 0.001.*

## Discussion

In this study, we compared the recruitment niche of plants, inferred from a seed bank experiment, with the observed community composition across a hydrological gradient in plowed and undisturbed transects within a former agricultural wetland. Our results demonstrate that the recruitment niche determined from seed bank experiments is strongly correlated with community composition in the field across the plowed and undisturbed area. These results are consistent with assuming that species composition in hydrological gradients is either dominantly driven by the recruitment niche, or that recruitment and adult niche are very similar for these wetland plant communities. The fact that community composition in plowed areas was slightly more correlated with the predicted niche was expected likely due to the conditions between the seed bank and plowing being more analogous to one another, as the mixing of seeds in the seed bank experiment is comparable to what occurs after plowing. However, there was a surprisingly strong correlation between the plowed and undisturbed areas, with 80% of the species being shared.

The strong control of water level on community composition demonstrated in the seed bank experiment shows that hydrological conditions are of major importance for germination and establishment in the plant community considered in this study. The small differences between different submergence depths is in accordance with previous seed bank studies that have found water levels to influence seedling emergence (Baldwin et al., 2001; Peterson and Baldwin, 2004; Facelli et al., 2005), abundance (van der Valk and Davis, 1978), survival and growth (Fraaije et al., 2015b), species richness (Keddy and Reznicek, 1986; Seabloom et al., 1998; Xiong et al., 2003), and community composition (Marañón, 1998; Casanova and Brock, 2000; Nicol et al., 2003; dos Santos et al., 2013). The germination niche of amphibious plants which may grow as adults under terrestrial and submerged conditions such as *Alisma* spp., *Eleocharis palustris*, *Juncus* spp. or *Veronica* spp. may be often less broad than the adult’s plant niche. *Alisma* spp. had its germination maximum in the seed bank experiment under permanently moist conditions, *Juncus* spp. and *Veronica* spp. either under permanently dry or moist but never under flooded conditions. In contrast, *Eleocharis palustris* germinated under all hydrological conditions. The difference between the recruitment and the adult niche of was already stated by Grubb (1977) and Young et al. (2005). The results also show that the soil seed bank composition has not always to represent the composition above-ground vegetation which is in contrast to most of the seed bank studies; e.g., for hayfield succession (Bekker et al., 2000) but may reflect either former land use types which were abandoned in former times (Karlík and Poschlod, 2014) or the fact of annually or seasonally changing habitat conditions as it is usual in amphibious habitats or artificial ponds occasionally drained in summer (Poschlod et al., 1999; Poschlod and Rosbakh, 2018).

These results have potential implications for the field of conservation and restoration ecology. The seasonal recruitment niche has been found to explain species composition of differently managed grasslands on the Swabian Jurassic Mountains (Kahmen et al., 2002; Kahmen and Poschlod, 2007; Drobnik et al., 2011). Moreover, anthropogenic disturbances may lead not only to changes in seed bank composition (Wisheu and Keddy, 1991; Devictor et al., 2007; Bart and Davenport, 2015), but potentially also increase biodiversity and activate previously “hidden” rare or threatened species (Poschlod and Rosbakh, 2018). Restoration success can, thereby, be improved by recognizing the recruitment niche for targeted species and influencing species composition through a hydrological gradient. Taken alongside with the knowledge of population dynamics, genetics, and other environmental factors, it may lead to more informed decisions in restoration ecology and improved outcomes in the recovery of formerly functional communities (Bakker et al., 2000).

A limitation of this study was that only forty percent of species observed in the field germinated in the seed experiment. This may simply be a result of sampling, which was much larger in the field than the seed experiment and may have reduced the number of seeds present in the soil samples. Another possibility is that since the soil was sampled in early spring, some species may have been represented with a transient seed bank and germination in autumn, which is the case for some dominant species such as *Phalaris arundinacea*, *Agrostis stolonifera* or *Salix spp.* (Thompson et al., 1997). Moreover, several wetland species also produce a low amount of (fertile) seeds (McKee and Richards, 1996; Leck and Schütz, 2005) or even reproduce only clonally, such as many members of Cyperaceae (Sosnová et al., 2010). However, the low proportion of germinating species could also be due to soil depth of the experiment, which may influence germination and community structure in wetland species (van der Valk and Davis, 1978; Casanova and Brock, 2000; Nicol et al., 2003; Faist et al., 2013). The soil experiment involved combining and mixing the soil to randomly distribute the seeds and may have resulted in species not being placed at their natural depth or in their optimum conditions. To improve such recruitment niche experiments, future studies may consider increasing samples from the seed bank either in autumn or earlier in the season or adding seeds for those species that produce a low amount of fertile seeds to determine their niche optimum.

This study supports the view that the recruitment niche is a crucial filter for community composition in the studied aquatic plant communities in both disturbed and undisturbed areas. We suggest that a better understanding of this filter in a plants life cycle may improve our understanding of species distributions and plant species assembly. Recognizing the importance of the recruitment niche in influencing species composition can be vital not just for ecological theory but for better predicting the effect of future environmental changes on plant communities, especially in the context of conservation and climate change.

## Author Contributions

JV wrote the manuscript, organized the database, and performed the statistical analysis. FH interpreted the data, advised regarding statistical methodology, and assisted with the manuscript. SF collected the seed experiment data. PP conceived and designed the experiment, collected the field experiment data, interpreted the data, and assisted with writing of the manuscript. All authors contributed to manuscript revision, read, and approved the submitted version.

## Conflict of Interest Statement

The authors declare that the research was conducted in the absence of any commercial or financial relationships that could be construed as a potential conflict of interest.
