# Supplementary material for: The Recruitment Niche Predicts Plant Community Assembly Across a Hydrological Gradient Along Plowed and Undisturbed Transects in a Former Agricultural Wetland
Source: Front Plant Sci. 2019 Feb 6;10:88. doi: 10.3389/fpls.2019.00088 (PMC6372561; doi:10.3389/fpls.2019.00088)
Supplement: Supplementary file 1 [file Data_Sheet_1.PDF]

Appendix S1. Values from the best GAM model predicting seed germination by species. Shape of the species response functions with confidence bands, optimum water level (1 = permanently dry, 2 = permanently moist, 3 = permanently flooded 10 cm above soil surface, and 4 = permanently flooded 40 cm above soil surface), and significance level (*P < 0.05, **P < 0.01, *** P < 0.001)

Species	Shape	Optimum	Significance level
Alisma lanceolatum	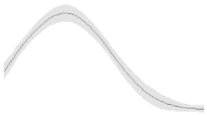	1.93	***
Alisma plantago-aquatica		n.s.	n.s.
Alisma spec.	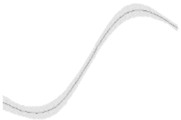	3.34	***
Bidens tripartita	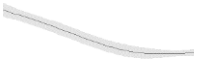	1	***
Chara spec.	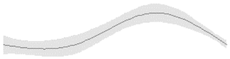	3.07	***
Chara vulgaris	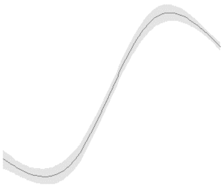	3.31	***
Eleocharis palustris	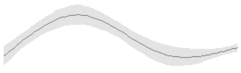	1.96	***
Epilobium ciliatum		1	*

Epilobium spec.	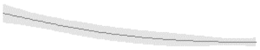	1	***
Euphorbia platyphyllos	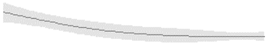	1	***
Galium palustre	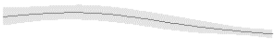	1.81	***
Juncus articulatus	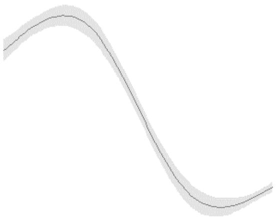	1.69	***
Juncus bufonius	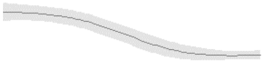	1	***
Juncus sphaerocarpus		n.s.	n.s.
Limosella aquatica	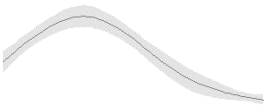	1.96	***

Lysimachia nummularia		n.s.	n.s.
Mentha arvensis	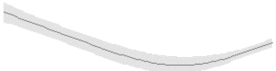	1	***
Persicaria lapathifolia	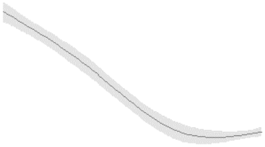	1	***
Persicaria maculosa	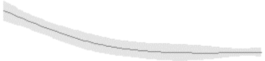	1	***
Phalaris arundinacea	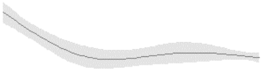	1	***
Plantago major	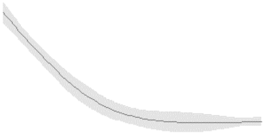	1	***
Poa trivialis	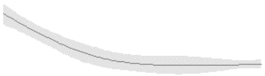	1	***

Ranunculus repens	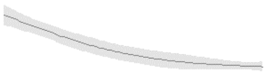	1	***
Rorippa palustris	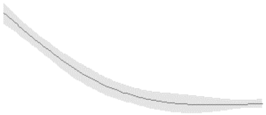	1	***
Rumex crispus		1	n.s.
Schoenoplectus supinus	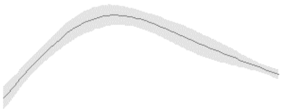	2.23	***
Scutellaria galericulata	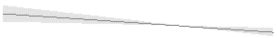	1	***
Sonchus arvensis	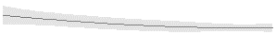	n.s.	n.s.
Sparganium erectum	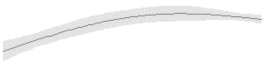	3.07	***

Stachys palustris	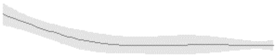	1	***
Trifolium repens		n.s.	n.s.
Typha latifolia	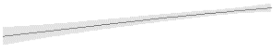	4	***
Veronica beccabunga		n.s.	n.s.
Veronica scutellata		n.s.	n.s.
